# Wedelolactone Enhances Osteoblastogenesis through ERK- and JNK-mediated BMP2 Expression and Smad/1/5/8 Phosphorylation

**DOI:** 10.3390/molecules23030561

**Published:** 2018-03-02

**Authors:** Di Zhu, Xue Deng, Xiao-Fei Han, Xiao-Xin Sun, Tao-Wen Pan, Lu-Ping Zheng, Yan-Qiu Liu

**Affiliations:** 1 Institute (College) of Integrative Medicine, Dalian Medical University, Dalian 116044, China; zhudi5256@163.com (D.Z.); dengxue222@163.com (X.D.); fly.sxx@163.com (X.-X.S.); pantw@dmu.edu.cn (T.-W.P.); zzllpp311@163.com (L.-P.Z.); 2 Glucose and Lipid Metabolism Laboratory of Liaoning Province, College of Life Science and Technology, Dalian University, Dalian 116622, China; helarpmi1640@126.com

**Keywords:** BMP2, MAPK, osteoblastogenesis, Smad, wedelolactone

## Abstract

Our previous study showed that wedelolactone, a compound isolated from *Ecliptae herba*, has the potential to enhance osteoblastogenesis. However, the molecular mechanisms by which wedelolactone promoted osteoblastogenesis from bone marrow mesenchymal stem cells (BMSCs) remain largely unknown. In this study, treatment with wedelolactone (2 μg/mL) for 3, 6, and 9 days resulted in an increase in phosphorylation of extracellular signal-regulated kinases (ERKs), c-Jun *N*-terminal protein kinase (JNK), and p38. Phosphorylation of mitogen-activated protein kinases (MAPKs), ERK and JNK started to increase on day 3 of treatment, and p38 phosphorylation was increased by day 6 of treatment. Expression of bone morphogenetic protein (BMP2) mRNA and phosphorylation of Smad1/5/8 was enhanced after treatment of cells with wedelolactone for 6 and 9 days. The addition of the JNK inhibitor SP600125, ERK inhibitor PD98059, and p38 inhibitor SB203580 suppressed wedelolactone-induced alkaline-phosphatase activity, bone mineralization, and osteoblastogenesis-related marker genes including Runx2, Bglap, and Sp7. Increased expression of BMP2 mRNA and Smad1/5/8 phosphorylation was blocked by SP600125 and PD98059, but not by SB203580. These results suggested that wedelolactone enhanced osteoblastogenesis through induction of JNK- and ERK-mediated BMP2 expression and Smad1/5/8 phosphorylation.

## 1. Introduction

Osteoblasts are derived from bone-marrow mesenchymal stem cell (BMSC) precursors [1], which differentiate into mature osteoblasts and mediate bone formation. This process is called osteoblastogenesis [2]. A deficiency in osteoblastogenesis of BMSCs can result in bone-related diseases including osteoporosis [3,4]. Reduced proliferation and osteoblastic differentiation of BMSCs has been reported to be associated with a reduction in healing capacity, such as impairment of bone formation in osteoporotic patients. Thus, developing drugs for targeting proliferation and osteoblastogenesis from BMSCs has become one therapeutic strategy for osteoporosis [5].

Wedelolactone is a compound isolated from *Ecliptae herba* [6,7,8]. This herbal drug has been used as a traditional Chinese medicine with kidney-tonifying acitivity for several thousand years because it can strengthen bones. *E. herba* extracts have been reported to exhibit therapeutic effects on osteoporosis in ovariectomized rats [9,10]. Wedelolactone has also been reported to possess various activities, including inhibiting the release of 5-lipoxygenase, antagonizing the action of myotoxins, and inducing caspase-dependent apoptosis. Previously, we showed that wedelolactone enhanced BMSC differentiation towards osteoblasts and promoted bone formation in vivo [11]. However, how wedelolactone enhances osteoblastogenesis is incompletely understood.

Bone morphogenetic protein (BMP) family proteins especially BMP2 are essential molecules that mediate osteoblastogenesis [12]. BMP2-mediated signaling is transduced through binding to its receptors and subsequently forming a receptor complex. This dynamic interaction transmits a signal through Smads or mitogen-activated protein kinases (MAPKs), leading to the activation of transcription of the specific target genes involved in osteoblastic differentiation and bone formation [13,14]. MAPK family members have been identified, including extracellular signal-regulated kinases (ERKs), c-Jun *N*-terminal protein kinase (JNK), and p38 [15,16]. These three types of MAPKs play essential roles in the signal-transduction machinery that participates in osteoblastogenesis [17,18]. During this osteoblastic differentiation process, activation of MAPK signaling can be followed by BMP2/Smad signaling activation [19].

We investigated how wedelolactone enhances osteoblastogenesis from BMSCs. In particular, the role of the MAPK/BMP2/Smads pathway-related molecules in wedelolactone-enhanced osteoblastogenesis was studied.

## 2. Results and Discussion

### 2.1. Effects of Wedelolactone on Phosphorylation of MAPK and Smad 1/5/8 and Expression of BMP2 mRNA 

**Figure 1:**
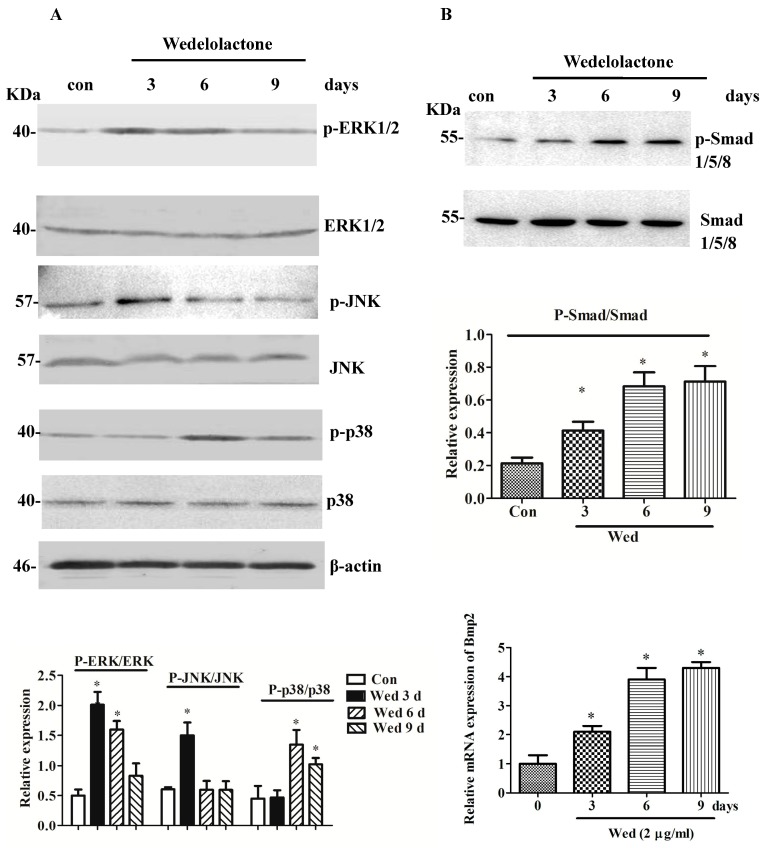
Effect of wedelolactone on phosphorylation of MAPK members, Smad1/5/8, and BMP2 mRNA expression in BMSC. (**A**) After the cells were treated with 2 μg/mL wedelolactone (Wed) for the indicated days, ERK phosphorylation increased at 3 days, and 6 days, JNK phosphorylation increased at 3 days, and p38 phosphorylation increased at 6 days and 9 days; (**B**) Smad1/5/8 phosphorylation increased upon 3 days, 6 days, and 9 days treatment; (**C**) BMP2 mRNA expression was measured by qRT-PCR and increased after 3 days, 6 days, and 9 days treatment. Data are expressed as mean ± SD of three independent experiments. * *p* < 0.05 compared with control.

To investigate if MAPK signaling was involved in wedelolactone-induced osteoblast differentiation, activation of MAPKs was examined by western blotting with specific antibodies. Wedelolactone (2 μg/mL) treatment activated ERK1/2, JNK, and p38, as evidenced by increased protein phosphorylation (Figure 1). Furthermore, the level of phosphorylated Smad1/5/8 was increased strongly upon 6-day treatment with wedelolactone. BMP2 expression was increased in the presence of 2 μg/mL of wedelolactone.

Wedelolactone is a compound isolated from *E. herba*, which has attracted considerable attention because of its extensive bioactivity [20]. Recently, we studied the effect of wedelolactone on osteoblastic differentiation from BMSCs [21]. However, the detailed signaling mechanisms involved in this effect are not known. In the present study, we focused on the effects of wedelolactone on osteoblastogenesis and associated bone-formation signaling including MAPK, Smads, and BMP2. At an effective dose, wedelolactone promoted phosphorylation of JNK, p38, and ERK1/2. In contrast, the phosphorylated levels of JNK, p38, and ERK1/2 did not show significant increase in osteogenic medium-treated BMSC without wedelolactone stimulation (data not shown). Wedelolactone-induced phosphorylation of ERK and JNK started from 3 days, and p38 phosphorylation started from 6 days. In contrast, TNFα or IL-1β-induced MAPK phosphorylation occurred within 24 h [22]. The difference might be attributed to a slow stimulation of wedelolactone. The delayed phosphorylation of p38 induced by wedelolactone suggested that p38 might mediate a different pathway for osteoblastogenesis compared with ERK and JNK. 

The results also revealed that wedelolactone stimulated osteoblast differentiation through Smad 1/5/8 and BMP2 signaling. These results suggested that MAPKs activation, Smad 1/5/8 phosphorylation, and increased BMP2 expression were involved in wedelolactone-enhanced osteoblastogenesis.

BMP2 signaling pathways have a critical role in bone-formation process, and their effects can be mediated by Smad signaling [23]. Among BMP family proteins, BMP2 has been reported to be essential for inducing bone formation [22]. Addition of BMP2 to culture media results in rapid proliferation of isolated mouse skeletal stem cells [24]. During wedelolactone-induced osteoblastogenesis, Smad 1/5/8 phosphorylation was enhanced and expression of BMP2 transcripts increased, suggesting that BMP2 and Smad1/5/8 participated in wedelolactone-induced osteoblastogenesis. Among 20 homodimeric or heterodimeric BMP ligands, there are many types of BMPs, which are indicated to be involved in osteoblastogenesis, such as BMP2, BMP3, BMP4, BMP6, BMP7, and BMP9 [25,26] We mainly focused on the effect of wedelolactone on the signaling pathway mediated by BMP2, one of the most well-known regulators for osteoblastogenesis. The further study of other BMPs changes will be interesting in the future. BMP2 promote bone formation through activating Smads. Smad1 is an important mediator of the osteogenic function of BMP2. In osteoblast-specific Smad1-conditional knockout (CKO) mice, BMP2 signaling is partially inhibited [27]. In addition to Smad1, Smad5 and Smad8 have also been implicated to be involved in BMP-mediated bone formation. Consistent with our previous results, the activation of BMP2 and Smad1/5/8 by wedelolactone in the present study suggested that the BMP2/Smad pathway probably was involved in wedelolactone-induced osteoblastogenesis in BMSCs.

Our previous study showed that wedelolactone facilitated osteoblastogenesis by inducing the Wnt/GSK3β/β-catenin pathway [28]. In the present study, MAPK signaling (another key pathway for osteoblastogenesis) was found to be induced. There are some crosstalking molecules between the Wnt/β-catenin pathway and MAPK pathway, such as Runx2. Identifying these crosstalking molecules triggered by wedelolactone would be interesting at the mechanistic level.

### 2.2. Effect of a MAPK Inhibitor on Wedelolactone-Enhanced ALP Activity and Bone Mineralization

**Figure 2:**
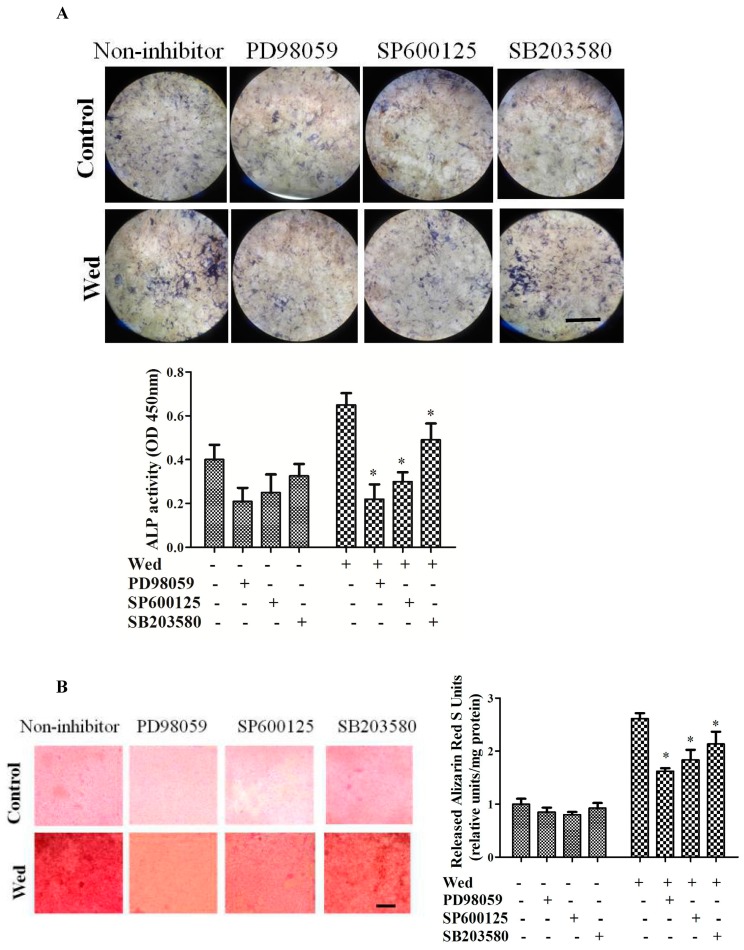
Effect of MAPK inhibitors on ALP activity and mineralization level. (**A**) BMSC pretreated with SP600125 (10 μM), PD98059 (20 μM), or SB203580 (10 μM) for 30 min were cultured in the presence of 2 μg/mL wedelolactone (Wed). Wedelolactone-induced increase in ALP activity was decreased by PD98059, SP600125, or SB203580. Scale bar is 100 μm; (**B**) Mineralization level elevated by wedelolactone were decreased after the cells were pretreated with PD98059, SP600125, or SB203580 as assayed by alizarin red staining. Scale bar is 100 μm. Data are expressed as mean ± SD of three independent experiments. * *p* < 0.05 compared with control.

To determine whether MAPK has a role in wedelolactone-induced osteoblastogenesis in BMSCs, specific inhibitors of ERK, JNK, and p38 were administered (Figure 2). Addition of the ERK inhibitor PD98059, JNK inhibitor SP600125, and p38 inhibitor SB203580 reversed the effect of wedelolactone on ALP activity to different degrees. Similarly, bone mineralization by Alizarin Red staining revealed that wedelolactone-enhanced bone mineralization was inhibited by PD98059, SP600125, and SB203580.

### 2.3. MAPK Inhibitors Prevent mRNA Expression of Wedelolactone-Induced Osteoblast Marker Genes

**Figure 3:**
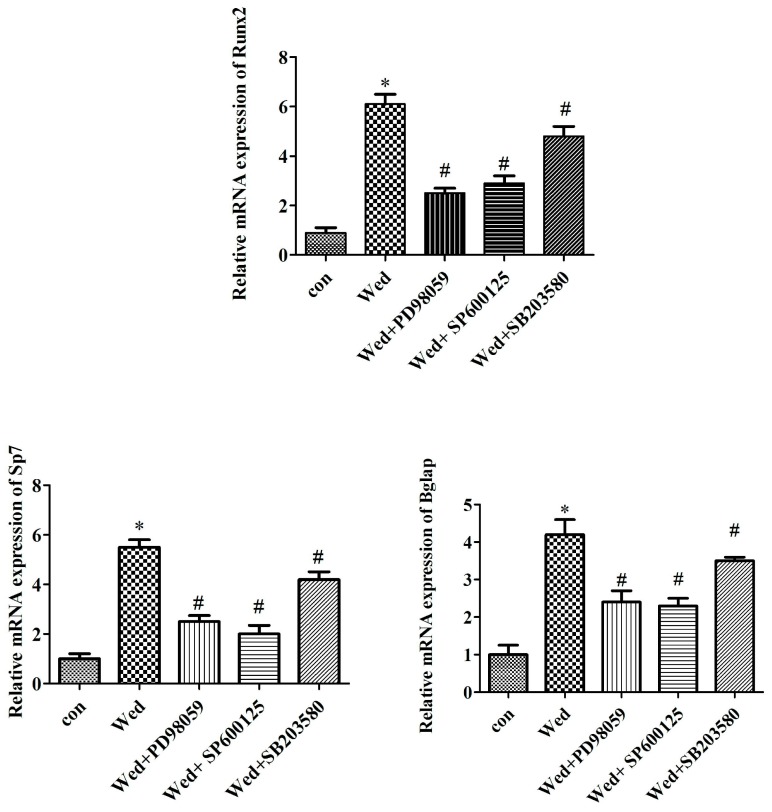
Effect of MAPK inhibitors on Runx2, Bglap, and Sp7 mRNA expression. BMSC were treated with SP600125 (10 μM), PD98059 (20 μM), or SB203580 (10 μM) for 30 min prior to 2 μg/mL wedelolactone (Wed) treatment for 9 days. Total mRNA was extracted from BMSC and qPCR was performed. Data are expressed as mean ± SD of three independent experiments. * *p* < 0.05 compared with control; # *p* < 0.05 compared with Wed-treated group.

We further examined whether the effect of wedelolactone on several osteoblast markers was mediated by MAPK signaling. mRNA expression of Runx2, Bglap (encoding osteocalcin), and Sp7 (which encodes osterix) were evaluated in BMSCs treated with specific inhibitors of JNK, ERK, and p38. Wedelolactone-induced increases in the expression of Runx2, Bglap, and Sp7 were attenuated by an ERK inhibitor, JNK inhibitor, and p38 inhibitor (Figure 3).

Several studies have reported that MAPK signaling including JNK, ERK, and p38 pathways are involved in osteoblastogenesis [29]. p38 is required for osteoblast differentiation and induction of osteogenic marker genes. Also, p38 activation has been observed in Lactoferrin-treated MC3T3-E1 cells. However, there is evidence that osteoblast differentiation is stimulated by activation of ERK and JNK, but not by activation of p38 [30]. In the present study, we demonstrated that inhibitors of JNK, ERK, and p38 suppressed wedelolactone-induced ALP activity, mineralization, as well as expression of osteoblast marker genes. These results are similar with a previous study to that of Kim and colleagues. They showed that activation of JNK and ERK was involved in osteoblast differentiation from human mesenchymal stem cells treated by fucoidan (a polysaccharide containing substantial proportions of l-fucose and sulfate ester groups that is derived mainly from brown algae and seaweed) [30]. The chemical structure of wedelolactone and fucoidan is different. Correspondingly, the targets for these two compounds are different but, interestingly, the wedelolactone- and fucoidan-induced signaling pathways including JNK and ERK were similar.

### 2.4. Activation of JNK and ERK Occurs Upstream of Smad 1/5/8 and BMP2 Signaling

**Figure 4:**
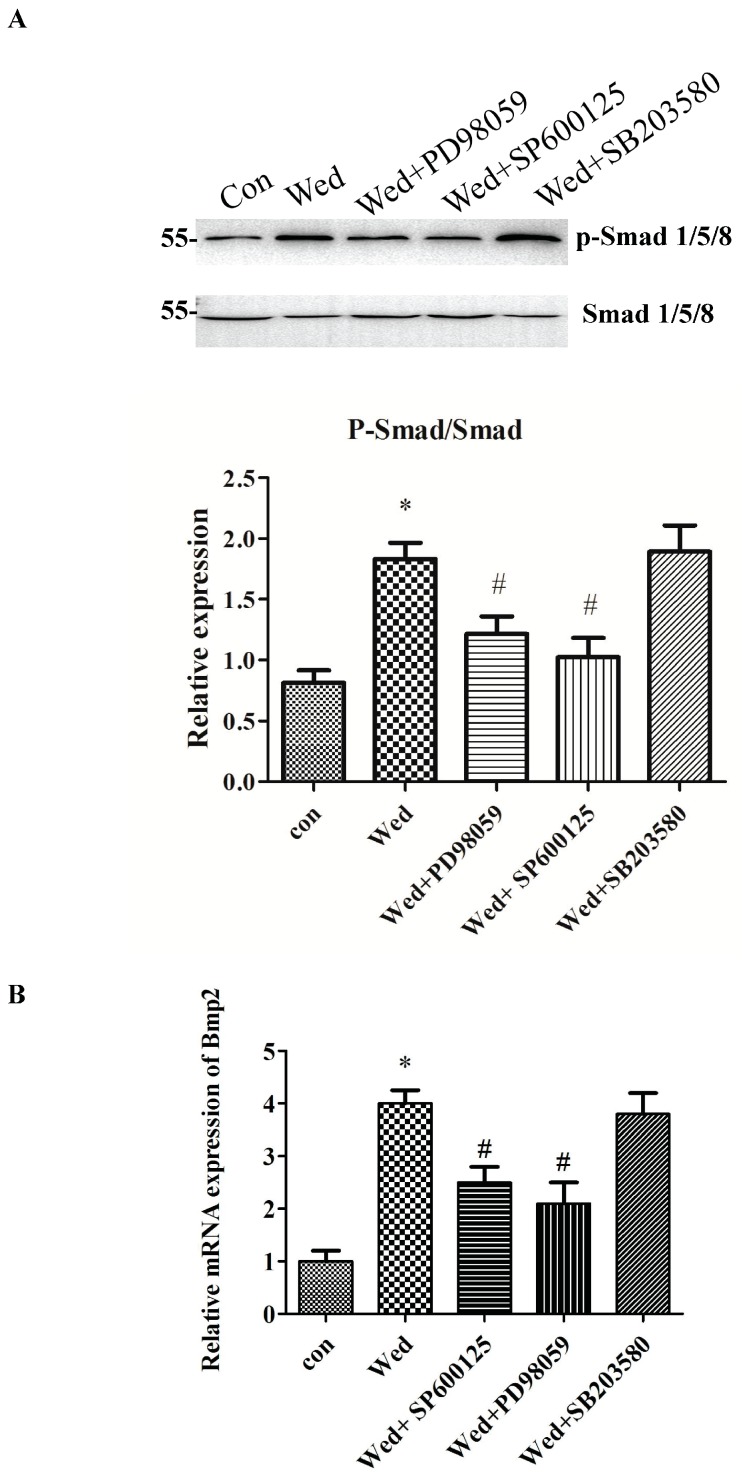
Effect of MAPK inhibitors on Smad1/5/8 phosphorylation and BMP2 mRNA expression. (**A**) BMSC were pretreated with MAPK inhibitors for 30 min, and then 2 μg/mL wedelolactone (Wed) was added to further culture for 6 days. Wedelolactone-induced Smad1/5/8 phosphorylation was decreased by PD98059 and SP600125; (**B**) Wedelolactone-increased BMP2 mRNA expression was inhibited by PD98059 and SP600125.Data are expressed as mean ± SD of three independent experiments. * *p* < 0.05 compared with control; # *p* < 0.05 compared with Wed-treated group.

To further evaluate whether Smad 1/5/8 or BMP2 signaling were mediated by MAPK, Smad 1/5/8 phosphorylation and BMP2 expression in cells were assayed after treatment with specific inhibitors of JNK, ERK, and p38. Addition of a JNK or ERK inhibitor prevented wedelolactone-induced phosphorylation of Smad 1/5/8 and BMP2 mRNA expression, whereas these effects were not observed in the presence of the p38 inhibitor (Figure 4). These data suggested that JNK and ERK acted upstream of BMP2 and Smad 1/5/8 in wedelolactone-induced osteoblastogenesis.

MAPK can act downstream as well as upstream of the BMP2 pathway. The formation of a complex of BMP2 and its receptors can phosphorylate MAPK p38 and ERK. BMP2 has been implicated to induce Runx2 activity by activating MAPK p38, ERK, and JNK. Conversely, activated JNK and ERK influence BMP2 expression [31]. Inhibition of JNK and ERK by SP600125 and PD98059 reduced BMP2 mRNA expression, suggesting that wedelolactone-induced BMP2 was mediated by MAPK JNK and ERK. Smads act as a group of intracellular proteins, which are critical for transducing signals to the nucleus upon binding with BMP ligands. After activation of the BMP signaling cascade, Smads interact physically with MAPK ERK to regulate the transcription of target genes, and thereby induces osteoblast differentiation [31], suggesting that Smads act upstream of MAPK ERK. However, there is evidence that ERK, JNK, or p38 phosphorylates Smad1/5/8, thereby inducing expression of osteoblast differentiation genes. In the present study, an ERK inhibitor and JNK inhibitor but not p38 inhibitor suppressed phosphorylation of Smad1/5/8, suggesting that ERK and JNK acted upstream of Smad1/5/8 to enhance wedelolactone-induced osteoblastogenesis.

p38 has been reported to interact with the Wnt/β-catenin pathway at different levels. Wnt can activate p38 but p38 can reinforce the Wnt/β-catenin pathway simultaneously [32]. In the present study, although p38 phosphorylation was upregulated by wedelolactone, a p38 inhibitor did not affect phosphorylation of Smad1/5/8, suggesting that p38 did not participate in the BMP2/Smad1/5/8 pathway in wedelolactone-enhanced osteoblastogenesis. Further study of the role of p38 in wedelolactone-induced Wnt/β-catenin pathway will be interesting.

## 3. Materials and Methods

### 3.1. Materials

Wedelolactone was isolated from an extract of *E. herba* and high-performance liquid chromatography was done as described previously [33]. Wedelolactone purity was >98%. Antibodies against ERK, JNK, p38, p-ERK, p-JNK, p-p38, Smad1/5/8, and p-Smad1/5/8 were purchased from Cell Signaling Technology (Danvers, MA, USA). TRIzol^®^ Reagent was obtained from Invitrogen (Carlsbad, CA, USA). SP600125, PD98059, and SB203580 were purchased from Cell Signaling Technology. Staining kits for alkaline phosphatase (ALP) were obtained from Sigma Aldrich (Saint Louis, MO, USA).

### 3.2. Isolation and Culture of Mouse BMSCs

BMSCs were isolated as described previously with some modification [34,35]. Briefly, BMSCs were isolated from the bone marrow of 8-week-old BALB/c mice and collected by using gradient centrifugation of mesenchymal stem cell-specific gradient solutions (Tianjin Haoyang Biological Manufacture, Tianjin, China). Phosphate-buffered saline (PBS)-buffered bone-marrow cell fraction was placed on the top of a gradient solution and centrifuged at 340× *g* for 20 min at room temperature. The collection was washed with PBS. Cell samples were resuspended in alpha-minimum essential medium (Gibco, Grand Island, NY, USA) supplemented with 10% fetal calf serum, 100 U/mL of penicillin, and 100 μg/mL of streptomycin, and maintained with 5% CO_2_ in a humidified atmosphere at 37 °C. On day 3, the cell suspension was replaced with fresh complete medium. After culture for 6–7 days, 90% confluence was reached. These cell samples were used for further experiments.

### 3.3. Assay to Measure ALP Activity

To measure ALP activity, BMSCs were reseeded (1 × 10^4^ per cm^2^) and cultured with osteogenic medium (100 nM of dexamethasone, 1 mM of β-glycerophosphate, and 5 μM of l-ascorbic acid 2-phosphate). The culture medium was changed every 3 days. After culture for 9 days, Cells were fixed with 60% citrate buffered acetone for 30 s. Then, the fixed cells were washed with water three times and were further incubated with 100 μL phosphatase substrate solution containing 10 mM p-nitrophenylphosphate (pNPP) and 10 mM sodium tartrate in 50 mM citrate buffer (PH 9.5) at 37 °C for 1 h. After incubation, the enzyme reaction mixture was transferred to another plate and the reaction was stopped with 100 μL of 0.1 N NaOH. Absorbance at 405 nm was measured using an ELISA reader (Tecan, Switzerland). ALP staining was carried out with an ALP staining kit according to the manufacturer’s (Sigma Aldrich) instructions.

### 3.4. Alizarin Red Staining

Alizarin Red staining was performed according to a previously published method [36]. Briefly, BMSCs were reseeded (1 × 10^4^ per cm^2^) and cultured with osteogenic supplement. After 21 days, cells were fixed with 4% paraformaldehyde for 10 min, and washed twice with deionized water. Cells were then stained with 2% Alizarin Red (pH 4.1) for 10 min at room temperature. After the cells were washed thrice with deionized water, an orange-red color appeared, which indicated the intensity of calcium deposits. For quantification, the alizarin red S stained cultures were further incubated with 100 mM cetylpyridinium chloride for 1 h to solubilize and release calcium-bound alizarin red into solution [36].

### 3.5. Quantitative Real-Time Reverse Transcription-Polymerase Chain Reaction (qRT-PCR)

After BMSCs were treated with MAPK inhibitors for 30 min, 2 μg/mL wedelolactone (Wed) was added and then incubated for the indicated number of days. Cell samples were collected. All work was carried out in a designated PCR-clean area. RNA was extracted and isolated from cells using TRIzol Reagent (Gibco) according to manufacturer instructions. RNA was treated with DNAse (DNase I-RNase-Free™; Ambion, Boston, MA, USA) to remove contaminating DNA; 200 ng of total RNA was reverse-transcribed with oligodT primers using a High Capacity cDNA RT kit (Applied Biosystems, Foster City, CA, USA) in a 20 µL cDNA reaction, as specified by the manufacturer. For qRT-PCR, the template cDNA was added to a 20 µL reaction with SYBR™ GREEN PCR Master Mix (Applied Biosystems, Foster City, CA, USA) and 0.2 µM of primer. Amplification was done by using an ABI Prism 7000 (Applied Biosystems) system for 40 cycles under the following conditions: an initial denaturation of 95 °C for 10 min, plus 40 cycles of 95 °C for 15 s, then 60 °C for 1 min. Fold-changes were calculated relative to β-actin using the ^△△^Ct method for analyses of mRNA levels. 

The primer sets (forward and reverse, respectively) used were 5′-GTACGCCAACACAGTGCTG-3′ and 5′-CGTCATACTCCTGCTTGCTG-3′ for mouse β-actin: 5′-GCCGGGAATGATGAGAACTA-3′ and 5′-GGTGAAACTCTTGCCTCGTC-3′ for mouse Runx2; 5′-GCCATCACCCTGTCTCCTAA-3′ and 5′-GCTGTGGAGAAGACACACGA-3′ for mouse Bglap; 5′-GGAGGTTTCACTCCATTCCA-3′ and 5′-TAGAAGGAGCAAGGGGACAGA-3′ for mouse Sp7; 5′-TGACTGGATCGTGGCACCTC-3′ and 5′-CAGAGTCTGCACTATGGCATGGTTA-3′ for mouse BMP2.

### 3.6. Western Blotting

Cells were pretreated with MAPK inhibitors for 30 min and then incubated with 2 μg/mL wedelolactone (Wed) for indicated days. Cells were lysed using lysis buffer containing 10 mM Tris/HCl (pH 7.5), 150 mM NaCl, 2 mM ethylenediaminetetraacetic acid (EDTA), 1% (*v/v*) Triton X-100, 1 mM Na_3_CO_4_, 1 mM phenylmethane sulfonyl fluoride, and 0.1 mM aprotinin. Cells were centrifuged at 16,000× *g* for 30 min at 4 °C. The protein concentration in the cell lysates was determined using the Bradford protein assay. The same amount of total protein (5 μg) was loaded onto 3–8% sodium dodecyl sulfate-polyacrylamide gels (Invitrogen, Carlsbad, CA, USA) and electrophoresed under reducing conditions. Western blotting was done as described previously using the following antibodies: P-JNK, JNK, P-p38, p38, and Smad1/5/8 (Cell Signaling Technologies). P-ERK1/2 and ERK 1/2 (Santa Cruz Biotechnology)

### 3.7. Statistical Analyses

Statistical analyses were undertaken with SPSS v16.0 (IBM, Armonk, NY, USA). Data are the mean ± SD from at least three independent experiments. The Student’s *t*-test was used for comparisons between two groups, and one-way analysis of variance was used if more than two groups were compared.

## 4. Conclusions

Wedelolactone, as an active compound isolated from *E. herba*, showed obvious activity in enhancing osteoblastic differentiation and bone mineralization through ERK- and JNK-mediated BMP2 expression and Smad1/5/8 activation. Our study provides new insights into the mechanism of action of wedelolactone in osteoblastogenesis from BMSCs.

## References

[1] Krebsbach P.H., Kuznetsov S.A., Bianco P., Robev P.G. (1999). Bone marrow stromal cells: Characterization and clinical application. Crit. Rev. Oral Biol. Med..

[2] Tan J., Xu X., Tong Z., Lin J., Yu Q., Lin Y., Kuang W. (2015). Decreased osteogenesis of adult mesenchymal stem cells by reactive oxygen species under cyclic stretch: A possible mechanism of age related osteoporosis. Bone Res..

[3] Kiernan J., Davies J.E., Stanford W.L. (2017). Concise Review: Musculoskeletal Stem Cells to Treat Age-Related Osteoporosis. Stem Cells Transl. Med..

[4] Mostafavinia A., Dehdehi L., Ghoreishi S.K., Hajihossainlou B., Bayat M. (2017). Effect of in vivo low-level laser therapy on bone marrow-derived mesenchymal stem cells in ovariectomy-induced osteoporosis of rats. J. Photochem. Photobiol. B Biol..

[5] Antebi B., Pelled G., Gazit D. (2014). Stem cell therapy for osteoporosis. Curr. Osteoporos. Rep..

[6] Syed S.D., Deepak M., Yogisha S., Chandrashekar A.P., Muddarachappa K.A., D’Souza1 P., Agarwal1 A., Venkataraman B.V. (2003). Trypsin inhibitory effect of wedelolactone and demethylwedelolactone. Phytother. Res..

[7] Sarveswaran S., Gautam S.C., Ghosh J. (2012). Wedelolactone, a medicinal plant-derived coumestan, induces caspase-dependent apoptosis in prostate cancer cells via downregulation of PKCɛ without inhibiting Akt. Int. J. Oncol..

[8] Wagner H., Fessler B. (1986). In vitro 5-lipoxygenase inhibition by Eclipta alba extracts and the coumestan derivative wedelolactone. Planta Med..

[9] Cheng M., Wang Q., Fan Y., Liu X., Wang L., Xie R., Charlene C.H., Sun W. (2011). A traditional Chinese herbal preparation Er-Zhi-Wan, prevent ovarietomy-induced osteoporosis in rats. J. Ethnopharmacol..

[10] Zhang Z.G., Bai D., Liu M.J., Li Y., Pan J.H., Liu H., Wang W.L., Xiang L.H., Xiao G.G., Ju D.H. (2013). Therapeutic effect of aqueous extract from *Ecliptae herba* on bone metabolism of ovariectomized rats. Menopause.

[11] Liu Y.Q., Hong Z.L., Zhan L.B., Chu H.Y., Zhang X.Z., Li G.H. (2016). Wedelolactone enhances osteoblastogenesis by regulating Wnt/β-catenin signaling pathway but suppresses osteoclastogenesis by NF-κB/c-fos/NFATc1 pathway. Sci. Rep..

[12] Noël D., Gazit D., Bouquet C., Apparailly F., Bony C., Plence P., Millet V., Turgeman G., Perricaudet M., Sany J. (2004). Short-term BMP-2 expression is sufficient for in vivo osteochondral differentiation of mesenchymal stem cells. Stem Cells.

[13] Kakita A., Suzuki A., Ono Y., Miura Y., Itoh M., Oiso Y. (2004). Possible involvement of p38 MAP kinase in prostaglandin E1-induced ALP activity in osteoblastlike cells. Prostaglandin Leukot. Essent. Fatty Acid.

[14] Daigang L., Jining Q., Jinlai L., Pengfei W., Chuan S., Liangku H., Ding T., Zhe S., Wei W., Zhong L. (2016). LPS-stimulated inflammation inhibits BMP-9-induced osteoblastic differentiation through crosstalk between BMP/MAPK and Smad signaling. Exp. Cell Res..

[15] Suzuki A., Palmer G., Bonjour J.P., Caverzasio J. (1999). Regulation of alkaline phosphatase activity by p38 MAP kinase in response to activation of Gi protein-coupled receptors by epinephrine in osteoblast-like cells. Endocrinology.

[16] Zhang W., Guo H., Jing H., Li Y., Wang X., Zhang H., Jiang L., Ren F. (2014). Lactoferrin stimulates osteoblast differentiation through PKA and p38 pathways independent of lactoferrin’s receptor LRP1. J. Bone Miner. Res..

[17] Ding D., Xu H., Liang Q., Xu L., Zhao Y., Wang Y. (2012). Over-expression of Sox2 in C3H10T1/2 cells inhibits osteoblast differentiation through Wnt and MAPK signalling pathways. Int. Orthop..

[18] Fu L., Tang T., Miao Y., Zhang S., Qu Z., Dai K. (2008). Stimulation of osteogenic differentiation and inhibition of adipogenic differentiation in bone marrow stromal cells by alendronate via ERK and JNK activation. Bone.

[19] Niu Y.B., Kong X.H., Li Y.H., Fan L., Pan Y.L., Li C.R., Wu X.L., Lu T.L., Mei Q.B. (2015). Radix Dipsaci total saponins stimulate MC3T3-E1 cell differentiation via the bone morphogenetic protein-2/MAPK/Smad-dependent Runx2 pathway. Mol. Med. Rep..

[20] Zhang H., Xing W.W., Li Y.S., Zhu Z., Wu J.Z., Zhang Q.Y., Zhang W., Qin L.P. (2009). Effect of a traditional Chinese herbal preparation on osteoblasts and osteoclasts. Maturitas.

[21] Liu Y.Q., Han X.F., Bo J.X., Ma H.P. (2016). Wedelolactone Enhances Osteoblastogenesis but Inhibits Osteoclastogenesis through Sema3A/NRP1/PlexinA1 Pathway. Front. Pharmacol..

[22] Huang R.L., Yuan Y., Tu J., Zou G.M., Li Q. (2014). Opposing TNF-α/IL-1β- and BMP-2-activated MAPK signaling pathways converge on Runx2 to regulate BMP-2-induced osteoblastic differentiation. Cell Death Dis..

[23] Wang M., Jin H., Tang D., Huang S., Zuscik M.J., Chen D. (2011). Smad1 plays an essential role in bone development and postnatal bone formation. Osteoarthr. Cartil..

[24] Phimphilai M., Zhao Z., Boules H., Roca H., Franceschi R.T. (2006). BMP signaling is required for RUNX2-dependent induction of the osteoblast phenotype. J. Bone Miner. Res..

[25] Cheng H., Jiang W., MPhillips F., CHaydon R., Peng Y., Zhou L., Luu H.H., An N., Breyer B., Vanichakarn P. (2003). Osteogenic activity of the fourteen types of human bone morphogenetic proteins (BMPs). J. Bone Jt. Surg. Am..

[26] Hen B., Wei A., Whittaker S., Williams L.A., Tao H., Ma D.D., Diwan A.D. (2010). The role of BMP-7 in chondrogenic and osteogenic differentiation of human bone marrow multipotent mesenchymal stromal cells in vitro. J. Cell. Biochem..

[27] Ghosh-Choudhury N., Abboud S.L., Nishimura R., Celeste A., Mahimainathan L., Choudhury G.G. (2002). Requirement of BMP-2-induced phosphatidylinositol 3-kinase and Akt serine/threonine kinase in osteoblast differentiation and mad-dependent BMP-2 gene transcription. J. Biol. Chem..

[28] Hata K., Ikebe K., Wada M., Nokubi T. (2007). Osteoblast response to titanium regulates transcriptional activity of Runx2 through MAPK pathway. J. Biomed. Mater. Res. A.

[29] Kim B.S., Kang H.J., Park J.Y., Lee J. (2015). Fucoidan promotes osteoblast differentiation via JNK- and ERK-dependent BMP2-Smad 1/5/8 signaling in human mesenchymal stem cells. Exp. Mol. Med..

[30] Kawaki H., Kubota S., Suzuki A., Suzuki M., Kohsaka K., Hoshi K., Fujii T., Lazar N., Ohgawara T., Maeda T. (2011). Differential roles of CCN family proteins during osteoblast differentiation: Involvement of Smad and MAPK signaling pathways. Bone.

[31] Selvamurugan N., Kwok S., Alliston T., Reiss M., Partridge N.C. (2004). Transforming growth factor-beta 1 regulation of collagenase-3 expression in osteoblastic cells by cross-talk between the Smad and MAPK signaling pathways and their components, Smad2 and Runx2. J. Biol. Chem..

[32] Chen J.R., Lazarenko O.P., Wu X., Kang J., Blackburn M.L., Shankar K., Badger T.M., Ronis M.J. (2010). Dietary-induced serum phenolic acids promote bone growth via p38 MAPK/β-catenin canonical Wnt signaling. J. Bone Miner. Res..

[33] Liu Y.Q., Zhan L.B., Liu T., Cheng M.C., Liu X.Y., Xiao H.B. (2014). Inhibitory effect of *Ecliptae herba* extract and its component wedelolactone on pre-osteoclastic proliferation and differentiation. J. Ethnopharmacol..

[34] Zhang J.F., Li G., Chan C.Y., Meng C.L., Lin M.C., Chen Y.C., He M.L., Leung P.C., Kung H.F. (2010). Flavonoids of Herba Epimedii regulate osteogenesis of human mesenchymal stem cells through BMP and Wnt/β-catenin signaling pathway. Mol. Cell. Endocrinol..

[35] Egermann M., Goldhahn J., Schneider E. (2005). Animal models for fracture treatment in osteoporosis. Osteoporos. Int..

[36] Johnson K.S., Hashimoto S., Lotz M., Pritzker K., Goding J., Terkeltaub R. (2001). Up-regulated expression of the phosphodiesterase nucleotide pyrophosphatase family member PC-1 is a marker and pathogenic factor for knee meniscal cartilage matrix calcification. Arthritis Rheum..

